# Identification of the NADPH Oxidase 4 Inhibiting Principle of *Lycopus europaeus*

**DOI:** 10.3390/molecules23030653

**Published:** 2018-03-14

**Authors:** Silvia Revoltella, Giorgia Baraldo, Birgit Waltenberger, Stefan Schwaiger, Philipp Kofler, Julia Moesslacher, Astrid Huber-Seidel, Konrad Pagitz, Roland Kohl, Pidder Jansen-Duerr, Hermann Stuppner

**Affiliations:** 1 Institute of Pharmacy/Pharmacognosy and Center for Molecular Biosciences Innsbruck (CMBI), University of Innsbruck, 6020 Innsbruck, Austria; silvia.revoltella@uibk.ac.at (S.R.); hermann.stuppner@uibk.ac.at (H.S.); 2 Institute for Biomedical Aging Research and CMBI, University of Innsbruck, 6020 Innsbruck, Austria; giorgia.baraldo@uibk.ac.at (G.B.); kofler.philipp@gmail.com (P.K.); pidder.jansen-duerr@uibk.ac.at (P.J.-D.); 3 Cura Marketing GmbH, 6020 Innsbruck, Austria; julia.moesslacher@cura.co.at (J.M.); astrid.huber-seidel@cura.co.at (A.H.-S.); roland.kohl@cura.co.at (R.K.); 4 Institute of Botany, University of Innsbruck, 6020 Innsbruck, Austria; konrad.pagitz@uibk.ac.at

**Keywords:** *Lycopus europaeus*, NADPH oxidase 4, rosmarinic acid, anti-ageing, ROS

## Abstract

NADPH oxidase 4 (Nox4) has recently been implicated as driving force in cellular senescence. Thus, there is growing interest to develop Nox4 inhibitors, which might be valuable agents for cosmeceutical applications. Alpine plants represent a valuable source for the identification of novel bioactive natural products with anti-ageing effects, especially substances that protect plants against UV radiation, which is also known to contribute to the ageing of human skin. Therefore, the aim of this study was to identify novel Nox4 inhibitors from alpine plants. Within an initial screening of extracts of alpine plants on their ability to inhibit Nox4 activity in HEK cells, the methanolic extract of the subaerial parts of *Lycopus europaeus* showed a strong inhibition of Nox4 (81% chemiluminescence quenching) and a simultaneously high cell viability (91% vitality). Rosmarinic acid was isolated and identified as the major compound in this bioactive extract. It showed a dose dependent inhibitory activity on Nox4 with an IC_50_ of 1 µM. Moreover, it also showed a significant inhibitory activity on Nox2 in the low micromolar range, whereas no inhibition of Nox5 was detected. Further investigations confirmed that the observed effects of rosmarinic acid on Nox2 and Nox4 are real inhibitory activities, and not due to ROS scavenging effects. Therefore, *L. europaeus*, which we demonstrated to be a good source of rosmarinic acid, has great potential for usage in cosmeceutical products with anti-ageing activity.

## 1. Introduction

**Figure 1:**
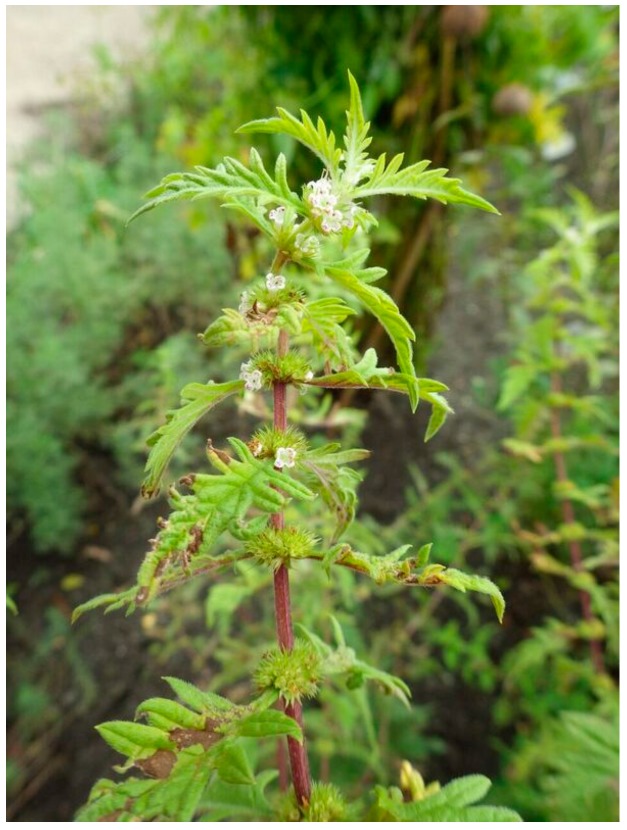
*Lycopus europaeus*.

The human Nox family comprises 7 enzymes, which are transmembrane proteins containing two heme groups involved in the transport of electrons from the cytosolic side through the membrane to produce reactive oxygen species (ROS). NADPH oxidase 4 (Nox4) is constitutively active and present in many cell types. It is involved in oxidative stress in different cell types like endothelial cells, smooth muscle cells, mesangial cells, adipocytes, and osteoclasts [1]. Recently, Nox4 was shown to be implicated in kidney diseases, such as diabetic nephropathy, and damage to the kidney induced by other pathways of renal damage, including advanced glycation end-products, the renin-angiotensin system, TGF-β, and protein kinase C. The role of Nox4 as a target for renoprotection remains controversial, although recent positive preclinical data have stimulated increased interest in inhibiting the enzyme in clinical trials of renal disease [2]. On the other hand, Nox4 may play a protective role in the kidney during renal ischemia reperfusion injury [3]. Several reports suggest that Nox4 plays an important role also in fibrotic diseases in several tissues. Thus, recent studies suggest that Nox4 mediated loss of cellular redox homeostasis promotes profibrotic myofibroblast phenotypes that result in persistent lung fibrosis associated with ageing. Restoration of the Nox4–Nrf2 redox balance in myofibroblasts may be a therapeutic strategy in age-associated fibrotic disorders of the lung, potentially able to resolve persistent fibrosis or even reverse its progression [4]. It has been proposed that pharmacological inhibition of Nox4 activity may represent a novel approach in the treatment of fibrotic disorders [5]. Furthermore, dysregulated redox homeostasis driven by elevated Nox4 derived ROS signaling underlies fibroblast-to-myofibroblast differentiation in the diseased prostatic stroma, indicating the potential clinical value of Nox4 inhibitors in preventing the functional pathogenic changes of stromal cells in benign prostate hyperplasia (BPH) and prostate carcinoma [6]. Nox4 derived oxidative stress is also considered as relevant pathomechanism of stroke, a leading cause of death and disability. Attenuating poststroke neurodegeneration by Nox4 inhibition has been proposed as a new concept in stroke therapy [7]. Finally, accumulating evidence suggests a role for Nox4 in skin ageing and skin diseases. Systemic sclerosis (SSc) is a systemic autoimmune disease characterized by progressive fibrosis of the skin and numerous internal organs [8]. Analyses of human dermal fibroblasts (HDFs) from patients with systemic sclerosis confirmed the expression of Nox4, whereas Nox1 and Nox2 were not detectable, indicating that Nox4 targeting is a promising future treatment for fibrotic skin diseases [9]. In ageing vasculature, Nox4 expression is upregulated and associated with smooth muscle cell polyploidy, a biomarker for ageing. ROS generating Nox4 is also a critical mediator of oncogene Ras-induced DNA damage and subsequent senescence [10]. Nox4 activity increases oxidative damage in human vascular endothelial cells, leading to cellular senescence [11]. Together, the available data suggest that Nox4 drives cellular ageing in several compartments of the skin, including the dermis and the microvasculature. Because of the pleiotropic role of Nox4 in many physiological and pathological processes, specific inhibitors of this enzyme may have a great therapeutic potential. Moreover, due to their expected ability to delay skin ageing, they might be valuable agents for cosmetic application. Focusing on this aspect of Nox4, we conducted an initial screening of 150 plants, mainly from the alpine region, which were collected, extracted, and investigated for their ability to inhibit Nox4 activity (data not shown). In general, plants which grow at high altitude and in hostile conditions contain often elevated levels of secondary metabolites that protect the plants against UV radiation damage [12,13], which is also known to contribute to the ageing of human skin [14]. Among the investigated plants, *Lycopus europaeus* L. (gypsywort; Figure 1), a perennial representative of the Lamiaceae family, was identified as one of the most promising plants, showing a high inhibitory activity against Nox4. 

*L. europaeus*, a plant native to Europe and Asia, is used in traditional medicine as an astringent, narcotic, and refrigerant [15]. Moreover, it is traditionally used in patients with slight hyperthyroidism and with vegetative-nervous disturbances [16]. This activity was recently confirmed in a study where a treatment with the extract of *L. europaeus* reduced the symptoms of hyperthyroidism in rats [17]. In addition to its medical usage, *L. europaeus* was also reported as a cosmetic agent [15]. Traditionally, the juice of the plant was used to produce a black dye used by gypsies (“gypsywort”) to tan their skin, supposedly to mimic Egyptians in Europe [18]. Moreover, the water extract of the leaves of the plant showed bactericidal activity on *Staphylococcus aureus*, which is one of the most frequent agents of skin infections. Therefore, *L. europaeus* could be considered a promising source of an antibacterial agent for topical usage [19]. 

The pharmacological effects of *L. europaeus* are often attributed to its phenolic compounds, e.g., rosmarinic and caffeic acid, which seem to be responsible for the biological activities [20,21,22]. Other compounds classes which have been reported to be present in the plant extracts are flavonoids (e.g., luteolin-7-*O*-glucuronide) [19], isopimarane-type diterpenoids, and alicyclic diterpenes [22], and other polyphenols, such as glucopyranosyl rosmarinic acid and sagerinig acid [19]. However, prior to this study, the effects of *L. europaeus* on Nox4 were still unknown, and therefore, within this study, extracts of this plant were investigated.

## 2. Results

### 2.1. Preparation of Extracts of L. europaeus and Investigation of Their NADPH Oxidase 4 (Nox4) Inhibitory Activities

A total amount of 5 g of dried aerial parts and 5 g of dried subaerial parts of *L. europaeus* were first extracted with ethyl acetate, followed by an extraction with methanol, respectively, in order to produce extracts of different polarity. The obtained extracts were tested for their ability to inhibit Nox4 by measuring Nox4-dependent chemiluminescence. In order to exclude false-positive results due to a low cell number, the extracts were additionally investigated in an MTT assay, measuring the cell viability upon sample treatment. The methanolic extract of the subaerial parts of the plant showed a strong inhibition of the Nox4 activity (81% chemiluminescence quenching), and at the same time, the lowest toxicity (91% vitality) (Table 1). Therefore, it was selected for further investigations.

### 2.2. Phytochemical Investigation of the Methanolic Extract of the Subaerial Parts of L. europaeus

**Figure 2:**
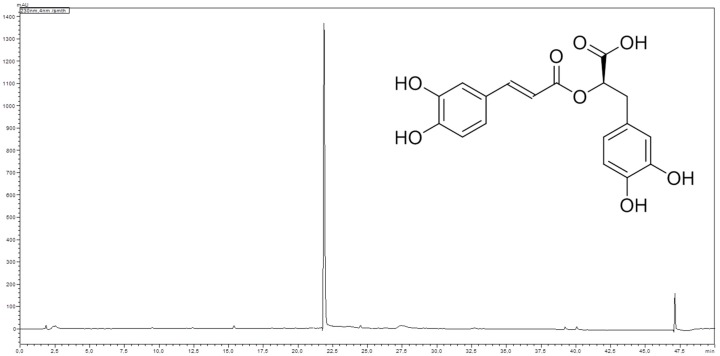
Chromatogram of the HPLC analysis of the methanol extract of the subaerial parts of *L. europaeus* at 230 nm. Analytical conditions: stationary phase: HyperClone 5 µm ODS (C18) 120 Å 150 × 4.6 mm; temperature: 30 °C; mobile phase: A = water + 0.02% trifluoroacetic acid, B = acetonitrile; flow rate: 1.00 mL/min; detection: 230 nm; injection volume: 10 µL; sample concentration: 5 mg/mL methanol; solvent composition during analysis: 0′: 2% B; 5′: 2% B; 35′: 50% B; 40′: 98% B; 45′: 98% B; 50′: 2% B.

Analysis of the methanolic extract of the subaerial parts of *L. europaeus* using high performance liquid chromatography (HPLC) revealed the presence of one major compound with UV max at 290 and 330 nm (Figure 2). Isolation of this major compound by Sephadex^®^ LH 20 column chromatography, followed by structure elucidation by means of LC-MS, 1- and 2D-NMR spectroscopy, and optical activity determination, as well as comparison of the spectral data with literature values [23], led to the identification of the major compound as rosmarinic acid (Figure 2).

### 2.3. Quantification of Rosmarinic Acid in L. europaeus Plant Material and Extracts

Since the investigated extracts showed divergent Nox4 inhibitory activities, we assumed that different contents of rosmarinic acid might be responsible for this discrepancy. Therefore, a HPLC method for the quantification of rosmarinic acid in different samples was established. Investigation of the different plant parts of the investigated sample of *L. europaeus* showed that the content of rosmarinic acid in the air dried aerial parts was found to be 1.96 ± 0.03 wt % (*n* = 2, analyzed in triplicate ± SD), while the air dried subaerial material contains 2.77 ± 0.04 wt % (*n* = 2, analyzed in triplicate ± SD).

Furthermore, the content of rosmarinic acid was also quantified in the investigated ethyl acetate and methanol extracts of the aerial and subaerial parts of *L. europaeus*, respectively. The obtained quantification results (Table 1) seem to confirm that a high rosmarinic acid content is connected to a high Nox4 inhibitory activity. However, due to the high differences in the corresponding cell viabilities, a final quantitative correlation cannot be drawn.

### 2.4. Effect of Rosmarinic Acid on Nox4

**Figure 3:**
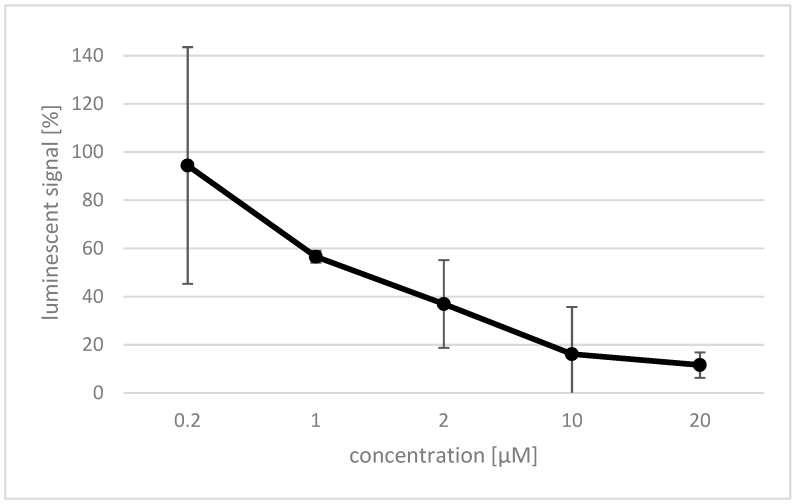
The effect of rosmarinic acid on Nox4 activity is dose-dependent. The amount of reactive oxygen species (ROS) produced by the HEK Nox4 cells and interaction with luminol were detected by luminometric measurement. The percentage of luminescent signal (*y*-axis) was established by comparison with an untreated control. Data shown as mean of triplicate ± SD.

Rosmarinic acid was investigated for its inhibitory effect on reactive oxygen species (ROS) production by Nox4. Therefore, the Nox4 gene was expressed in human embryonic kidney (HEK) cells [1], and a luminol based assay was established to detect ROS generated by the expressed protein. The effect of rosmarinic acid on HEK Nox4 cells was investigated using different concentrations between 0.2 and 20 µM of the compound, and was determined by a decrease in the luminescent signal (Figure 3). A concentration of 0.2 µM rosmarinic acid showed no significant effect. However, the effect rapidly increased at higher concentrations, and already, 20 µM rosmarinic acid completely quenched the measured luminescent signal. The IC_50_ value of rosmarinic acid was determined to be 1 µM.

### 2.5. Effect of Rosmarinic Acid on Nox2 and Nox5 Activity

**Figure 4:**
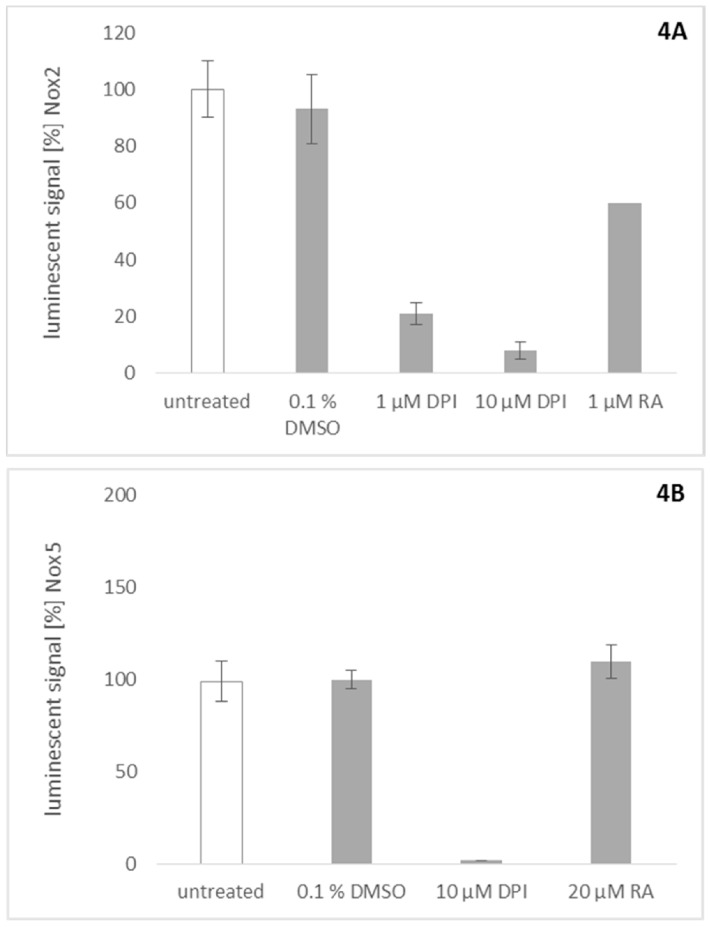

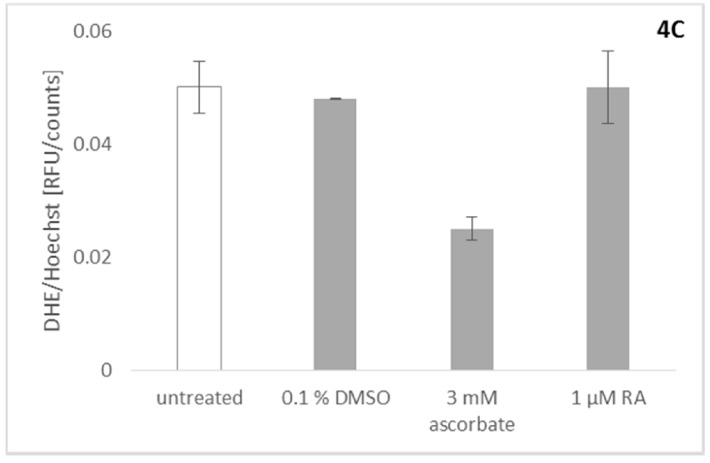
Effects of rosmarinic acid (RA) on Nox2 (**A**) and Nox5 (**B**) activity and control for ROS scavenging activity (**C**). Data are shown as mean (± SD) of nine independent experiments. (**A**) Rosmarinic acid was investigated for its inhibitory activity on Nox2. For the activation of Nox2, peripheral blood mononuclear cells (PBMCs) were treated with 2 ng/mL phorbol 12-myristate 13-acetate (PMA). Rosmarinic acid (1 µM) inhibits ROS production, as well as 1 µM diphenyleneiodonium (DPI); (**B**) Measuring the chemiluminescent signal on Nox5 overexpressing HEK cells after exposure to DPI a reduction of the luminescent signal was detected. However, concentrations up to 20 µM rosmarinic acid applied on the same cell system showed no effect, indicating that rosmarinic acid is not a universal Nox inhibitor; (**C**) Potential effects of rosmarinic acid on the ROS production by the mitochondrial respiratory chain have been investigated. For this approach, the respiratory chain was inhibited by the application of rotenone. The controls ascorbate and rotenone show a decrease and an unaffected mitochondrial ROS signal respectively onto U2OS. In its activity, rosmarinic acid resembles the negative control, showing no effect on mitochondrial ROS production.

Similar assays were used to probe rosmarinic acid for its activity towards Nox2 and Nox5. To investigate the effect of rosmarinic acid on Nox2, we stimulated peripheral blood mononuclear cells (PBMCs) with phorbol 12-myristate 13-acetate (PMA) to activate Nox2 ROS production. Rosmarinic acid showed significant inhibitory activity on Nox2 at a concentration of 1 µM (Figure 4A). This result is consistent with previous findings which show that many plant-derived Nox4 inhibitors also inhibit Nox2 activity in their effective concentration range [1]. To assess Nox5 activity, stably transfected Nox5 overexpressing HEK cells were stimulated with ionomycin [24]. This activity could be quenched completely by DPI (Figure 4B). However, up to a concentration of 20 µM, rosmarinic acid was inactive in this assay.

### 2.6. Effect of Rosmarinic Acid on Mitochondrial ROS Production

Since the activity of Nox4 in our assay cannot be measured directly, the products of the catalyzed reaction, ROS, have to be assayed. This necessitates discrimination between a real Nox4 inhibitor and a substance with merely ROS scavenging abilities [25]. To exclude the possibility that rosmarinic acid acts in the assayed concentration rage as a simple ROS scavenger, it was counterscreened for its ROS-scavenging ability. Therefore, we used an assay based on U2OS osteosarcoma cells stimulated to produce ROS by the addition of roteneone [1]. Rotenone is an inhibitor of complex I of the mitochondrial electron transport chain, which was shown to induce mitochondrial ROS [26]. Dihydroethidium (DHE) was used to determine the amount of ROS produced in the system. Briefly, DHE (blue fluorescence color) is oxidized by ROS to ethidium (red fluorescence color) which can, upon oxidation, intercalate into DNA, thereby drastically increasing the extinction coefficient and the fluorescence. To correct for the influence of toxic effects on cell numbers, cells were stained afterwards with Hoechst 33342. This was done to correct for dead or detached cells, since only viable cells would be stained, and only adherent cells would be detectable after the washing procedure. DHE signals were normalized with the factor obtained by the Hoechst 33342 assay, to correct for dead cells due to toxic effects [1]. The rotenone induced signal could be reduced completely by the addition of ascorbate, as was already described earlier [26]. Figure 4C shows that rosmarinic acid has no unspecific ROS scavenging ability in the concentration necessary to inhibit Nox2/4 activity.

## 3. Discussion

Rosmarinic acid is a phenolic compound which has been isolated from a variety of species of the Lamiaceae family [27], including *L. europaeus* [28]. Rather, high rosmarinic acid contents were found in *Mentha* species, especially in *M. spicata* (1.93–5.85 wt %), but also in *Salvia officinalis* (3.93 wt %) and *Thymus vulgaris* (2.35 wt %). *Rosmarinus officinalis* showed a slightly lower content of the compound (0.72 wt %) [29]. Our results revealed that the contents of rosmarinic acid in the aerial and subaerial parts of the investigated sample of *L. europaeus* are 1.96 wt % and 2.77 wt %, respectively. Therefore, *L. europaeus*, and especially the subaerial parts of the plant, can be considered as a valuable source of rosmarinic acid. Different pharmacological effects were already reported for rosmarinic acid, including antioxidative [30,31], anti-inflammatory [32], antimicrobial [33], antiviral [34], antirheumatic, antiallergic, antidiabetic [35], antidepressant [36], and antitumor [37] activities. Although the ability of rosmarinic acid to reduce oxidative stress in differentiated HL-60 cells has been reported [38], nothing is known regarding its ability to inhibit NOX enzymes, and its selectivity for the different members of this protein family. Our results showed that the effect of rosmarinic acid on Nox4 activity is dose dependent, and the IC_50_ was determined to be 1 µM. Furthermore, rosmarinic acid exhibited also significant inhibition of Nox2 at a concentration of 1 µM. This finding is consistent with previous findings which showed that many plant-derived Nox4 inhibitors also inhibit Nox2 in their effective concentration range [1]. On the other hand, rosmarinic acid did not show a significant inhibitory activity of Nox5 up to a concentration of 20 µM, and shows, therefore, a clear preference towards Nox2/4. An additional experiment to detect unspecific ROS scavenging abilities confirmed that the observed effects of rosmarinic acid at the investigated concentrations seem to really be the result of a specific Nox2/Nox4 inhibition. Since the aim of the study was the discovery of Nox4 inhibitors for cosmetic products with anti-ageing activity on skin cells (cosmeceuticals), the typical application route would be a topical formulation, like a cream or lotion. Since the effected cells of the upper skin do normally express the Nox4 isoform, the dual inhibition of Nox2/4 does not seem to be relevant for the investigated application, but might be of interest for other considerations, since rosmarinic acid is present in a variety of different spices and teas. As part of our daily diet, it might have an impact via this newly found Nox2/4 interaction on several pathologies and ROS mediated diseases. 

## 4. Materials and Methods

### 4.1. Chemicals and Solvents

All used solvents and chemical were purchased from VWR (Vienna, Austria), if not stated otherwise.

### 4.2. Plant Material

The plant material was collected at the Botanical Garden of the University of Innsbruck, Austria, on 8 July 2014. It was air-dried and a voucher specimen (BW-20140708-18) is stored at the Institute of Pharmacy/Pharmacognosy of the University of Innsbruck.

### 4.3. Extract Preparation for Isolation and Pharmacological Investigations

From the dried and grounded aerial and subaerial parts of *L. europaeus*, ethyl acetate, and methanol extracts were prepared, respectively. In particular, 5 g of dried and grounded plant material was extracted by sonication for 10 min with 30 mL of ethyl acetate, and centrifuged for 5 min at 2800 rpm and 25 °C. The supernatant was collected, and the same procedure was repeated twice using 20 mL of ethyl acetate, respectively. The supernatants were combined, and the solvent was evaporated to dryness to yield the crude extract. The plant material was dried and re-extracted with methanol following the same procedure.

### 4.4. Isolation and Structure Elucidation

The obtained methanol extract of the subaerial parts (175 mg) was subjected to Sephadex^®^ LH-20 (GE Healthcare, Uppsala, Sweden) column chromatography (560 mm × 10 mm) using water/methanol (1 + 3, *v*/*v*) as mobile phase. The eluate was collected in portions of 2 mL, which were combined according to their composition monitored by thin layer chromatography (TLC) (silica gel 60 on aluminum, layer 0.2 mm, F_254_, No. 5554, Merck, Darmstadt, Germany) using ethyl acetate/methanol/water/formic acid (50 + 10 + 7 + 1; *v*/*v*/*v*/*v*) as mobile phase and UV detection at 254 nm. Six fractions were obtained. Fraction 5 (12 mg), a yellow amorphous solid, comprised the main compound, isolated with a purity of 95.07% at 330 nm and 96.58% at 190 nm, according to HPLC analysis. HPLC-ESI-MS analyses were performed on an 1100 Agilent system (Agilent, Waldbronn, Germany) using the following analytical conditions: stationary phase: Phenomenex HyperClone 5 µm ODS (C18) 120 Å (150 mm × 4.6 mm); mobile phase: water bidestilled containing 0.9% formic acid and 0.1% acetic acid (A) and acetonitrile (B); solvent composition during analysis: 0′: 98% A; 5′: 98% A; 35′: 50% A; 40′: 2% A; 45′: 2% A; 50′: 98% A; flow rate: 1.00 mL/min; injection volume: 10 µL; temperature: 30 °C; detection: 230 nm. HPLC was hyphenated via 1:5 split to an Esquire 3000 plus (Bruker Daltonics, Bremen, Germany) ion trap using ESI in alternating mode with the following settings: temperature: 350 °C; dry gas: 10.00 L/min; nebulizer: 40 psi N_2_; full scan mode: *m*/*z* 100–1500. The major compound showed in following ions: positive mode *m*/*z* 361.2 (13.17%) [M + H]^+^ and 743.3 (8.01%) [2M + Na]^+^, negative mode *m*/*z* 359.4 (6.60%) [M − H]^−^, 473.1 (100%) [M − H + TFA]^−^, and 718.9 (4.86%) [2M − H]^−^. ^1^H- and ^13^C-NMR spectra of the isolated compound were recorded in methanol-d_4_ at a Bruker Avance II 600 spectrometer (Bruker) operating at 600.19 MHz (^1^H) and 150.91 MHz (^13^C) giving the following values: ^1^H-NMR (600.19 MHz, CD_3_OD) δ_H_ 7.50 (d, *J* = 15.9 Hz, 1H, H-7), 7.03 (d, *J* = 2.0 Hz, 1H, H-2), 6.92 (dd, *J* = 8.2, 1.9 Hz, 1H, H-6), 6.78–6.75 (m, 2H, H-5 and H-2′), 6.67 (d, *J* = 8.0 Hz, 1H, H-5), 6.63 (dd, *J* = 8.1, 1.9 Hz, 1H, H-6′), 6.27 (d, *J* = 15.9 Hz, 1H, H-8), 5.07 (dd, *J* = 9.8, 3.3 Hz, 1H, H-8′), 3.09 (dd, *J* = 14.3, 3.3 Hz, 1H, H-7′), 2.93 (dd, *J* = 14.3, 9.8 Hz, 1H, H-7′); ^13^C-NMR (150.91 MHz, CD_3_OD) δ_C_ 128.02 (C-1), 115.12 (C-2), 146.72 (C-3), 149.35 (C-4), 116.46 (C-5), 122.86 (C-6), 146.56 (C-7), 115.73 (C-8), 169.12 (C-9), 131.27 (C-1′), 117.50 (C-2′), 145.95 (C-3′), 144.79 (C-4′), 116.19 (C-5′), 121.74 (C-6′), 38.83 (C-7′), 77.85 (C-8′), 177.67 (C-9′). The obtained NMR spectra are shown in the Supplementary Materials Figures S1–S4. The optical rotation was determined with a Perkin-Elmer 341 polarimeter (Wellesley, MA, USA) at 20 °C showing an optical activity of $\alpha_{20}^{D}$ + 106.08 (*c* = 0.115 g/100 mL; methanol).

### 4.5. Quantification of Rosmarinic Acid in Aerial and Subaerial Parts of L. europaeus and Dry Extracts

Dried aerial parts of *L. europaeus* were cut into pieces and ground to a fine powder. Dried subaerial parts were cut, ground, and sieved (750 µm mesh; size IV, ÖAB, VWR, Vienna, Austria) in order to remove larger fibers. The plant material (30.0 mg) was extracted five times with 1.0 mL of methanol by sonication (10 min each, at ambient temperature) and then centrifuged at 5000 rpm for 5 min at 25 °C. Supernatants were combined, quantitatively transferred to a volumetric flask, and adjusted to the final volume (5.0 mL) with methanol. Prior to injection, all solutions were filtered through cotton wool. Each sample solution was assayed in triplicate. The exhaustiveness of the extraction procedure was assessed by HPLC analysis of an additional extraction step, which showed no detectable amounts of rosmarinic acid. Quantification of RA in extracts was performed by dissolving 4.00 mg of the ethyl acetate extracts, as well as 2.00 mg of the methanolic extracts in 1.00 mL methanol. Solutions were prepared in duplicate, filtered over cotton wool, and analyzed in triplicate. HPLC analyses were performed on a Shimadzu UFLC-XR instrument (Kyoto, Japan) equipped with auto-sampler, PDA, online degasser, and column thermostat using the following analytical conditions: stationary phase: Phenomenex HyperClone 5 µm ODS (C18) 120 Å (150 mm × 4.6 mm); mobile phase: water bidestilled containing 0.02% trifluoroacetic acid (A) and acetonitrile (B); solvent composition during analysis: 0′: 98% A; 5′: 98% A; 35′: 50% A; 40′: 2% A; 45′: 2% A; 50′: 98% A; flow rate: 1.00 mL/min; injection volume: 10 µL; temperature: 30 °C detection: 330 nm. For external calibration, two stock solutions of the isolated rosmarinic acid dissolved in methanol were prepared, diluted to 500, 250, 100, and 50 µg/mL, and analyzed in triplicate. Calibration curve was constructed by linear regression of analyte concentration against peak area at 330 nm, while the limit of detection (LOD) and the limit of quantification (LOQ) were calculated from the standard deviation of the response and the slope of the calibration curve following the ICH guidelines Q2 (R1) [39] resulting in the following parameters: *y* = 11904.11*x* − 7719.99, R^2^: 0.9992, LOD: 16.81 µg/mL, and LOQ: 56.05 µg/mL.

### 4.6. Cell Viability Assays

#### 4.6.1. MTT Assay

The MTS and PMS solutions for the MTT assay were purchased from Promega. The standard protocol suggested by Promega was applied. An incubation period of two hours was chosen for HEK Nox4 and HEK pcDNA cells as optimized condition (data not shown). The measurement was performed at 490 nm using a Tecan Infinite M200 instrument (Tecan Trading AG, Männedorf, Switzerland).

#### 4.6.2. Hoechst 33342 Staining

Alternatively, cell permeable Hoechst 33342 staining was performed to correct for detached or dead cells. Only viable cells would be stained by Hoechst 33342 and only adherent cells were measured [40,41], and the signal was not disturbed, nor did it interfere with the DHE signal. Experiments were performed either on a Victor X5 plate reader (excitation at 355 nm and emission at 460 nm; Perkin Elmer, Waltham, MA, USA) or an Infinite M200 instrument (excitation at 355 ± 9 nm and emission at 465 ± 20 nm; Tecan Trading AG, Männedorf, Switzerland), as described [1].

### 4.7. Nox4 Assay

HEK Nox4 and HEK pcDNA cells were seeded in 96 well plates and incubated overnight to allow the cells to attach to the surface. For the analysis of crude extracts and pure compound, 40,000 cells and 5000 cells were seeded in each well, respectively. The subsequent day, the cells were exposed to 125 µg/mL of the plant extract or different concentrations of the pure compound for one hour. After incubation, the cells were washed using Hank’s Balanced Salt Solution (HBSS) to remove any plant residues. Subsequently, luminol solution was applied and the chemiluminescence signal was measured using the Victor X5 plate reader (Perkin Elmer, Waltham, MA, USA). The luminescent signal of HEK Nox4 cells was normalized with the HEK pcDNA cells’ signal, which was interpreted as the basal ROS level produced.

### 4.8. Nox2 Assay

Nox2 chemiluminescence was assessed on PBMCs in suspension. The cells were centrifuged, washed in HBSS, and resuspended in HBSS based chemiluminescence mixture at an amount of 10,000 cells per well. Nox2 activity in PBMCs was activated by the addition of phorbol 12-myristate 13-acetate (PMA) to a final concentration of 2 µg/mL, and the chemiluminescence signal was measured using a Victor X5 plate reader (Perkin Elmer, Waltham, MA, USA) as described [1].

### 4.9. Nox5 Assay

Nox5 chemiluminescence was performed on adherent Nox5 transfected HEK 293 cells compared to empty pcDNA vector cells, controlled by diphenyleneiodonium (DPI). Nox5 transfected HEK 293 FT cells were seeded out one day prior to the experiment at 90,000 cells per well, to keep them dense for the experiment. Cells were checked for confluency under the microscope on the day of the experiment. Ionomycin was added to a final concentration of 1 µM, and the chemiluminescence signal was measured using a Victor X5 plate reader (Perkin Elmer, Waltham, MA, USA), as described [1].

### 4.10. Mitochondrial ROS Assay

Nox5 U2OS cells were cultivated in black 96-well plates, flat bottom, optical bottom, tissue culture treated, at 50,000 cells per well. Cells were seeded out one day prior to the experiment, and confluency was controlled on the day of the experiment. At first, the cells were washed carefully with pre-warmed HBSS to remove residues of the medium containing FCS, since this would influence the assay. In order to reduce washing steps, DMEM medium without phenol red and without FCS was prepared, containing 0.2 µM rotenone and 20 µM DHE, and incubated on the cells in the presence of the pure compound for 30 min at 37 °C. The staining solution was then removed, and the cells were measured in HBSS containing 5 µg/mL Hoechst 33342, which did not disturb the experiment. DHE signals were measured either on a Victor X5 plate reader (excitation at 531 ± 25 nm, emission at 595 ± 60 nm; Perkin Elmer, Waltham, MA, USA) or an Infinite M200 instrument (excitation at 510 ± 9 nm and emission at 590 ± 20 nm; Tecan Trading AG, Männedorf, Switzerland), as described [1].

## Figures and Tables

**Table 1:** Effects of extracts of *L. europaeus* on Nox4 inhibitory activity and cell viability (mean of two independent experiments, each performed in triplicate) and rosmarinic acid content of the extracts (*n* = 2, analyzed in triplicate ± SD).

Plant Part	Extraction Solvent	Extract Yield (wt %)	Nox4-Dependent Chemiluminescence Quenching (%) at 125 µg/mL	Cell Viability (%) at 125 µg/mL	Rosmarinic Acid Content (wt %)
subaerial parts	ethyl acetate	0.98	30 ± 8.90	63 ± 0.043	1.02 ± 0.003
subaerial parts	methanol ^1^	3.60	81 ± 5.07	91 ± 0.004	10.14 ± 0.120
aerial parts	ethyl acetate	2.50	98 ± 0.41	15 ± 0.003	0.26 ± 0.001
aerial parts	methanol ^1^	3.52	79 ± 4.77	69 ± 0.010	7.50 ± 0.140

^1^ after ethyl acetate extraction.

## Supplementary Materials

Supplementary materials are available online. Figures S1–S4: ^1^H-NMR; HSQC-NMR, HMBC-NMR and COSY-NMR-spectra of rosmarinic acid.
