# Supplementary material for: Identification of the NADPH Oxidase 4 Inhibiting Principle of Lycopus europaeus
Source: Molecules. 2018 Mar 14;23(3):653. doi: 10.3390/molecules23030653 (PMC6017630; doi:10.3390/molecules23030653)

Supplementary Materials

Identification of the NADPH oxidase 4 inhibiting principle of *Lycopus europaeus*

Silvia Revoltella [1], Giorgia Baraldo [2], Birgit Waltenberger [1,*], Stefan Schwaiger [1,*], Philipp Kofler [2], Julia Moesslacher [3], Astrid Huber-Seidel [3], Konrad Pagitz [4], Roland Kohl [3], Pidder Jansen-Duerr [2], and Hermann Stuppner [1]

[1] Institute of Pharmacy/Pharmacognosy and Center for Molecular Biosciences Innsbruck (CMBI), University of Innsbruck, 6020 Innsbruck, Austria; silvia.revoltella@uibk.ac.at (S.R.); birgit.waltenberger@uibk.ac.at (B.W.); stefan.schwaiger@uibk.ac.at (S.S.); hermann.stuppner@uibk.ac.at (H.S.)
[2] Institute for Biomedical Aging Research and CMBI, University of Innsbruck, 6020 Innsbruck, Austria; giorgia.baraldo@uibk.ac.at (G.B.); kofler.philipp@gmail.com (P.K.); pidder.jansen-duerr@uibk.ac.at (P.J.-D.)
[3] Cura Marketing GmbH, 6020 Innsbruck, Austria; julia.moesslacher@cura.co.at (J.M.); astrid.huber-seidel@cura.co.at (A.H.S.); roland.kohl@cura.co.at (R.K.)
[4] Institute of Botany, University of Innsbruck, 6020 Innsbruck, Austria; konrad.pagitz@uibk.ac.at (K.P.)
* Correspondence: birgit.waltenberger@uibk.ac.at; Tel.: +43-512-507-58420; stefan.schwaiger@uibk.ac.at; Tel.: +43-512-507-58409

Spectra of Rosmarinic acid

Figure S1: ^{1}H-NMR spectrum (600.19 MHz) of rosmarinic acid in CD_3OD.

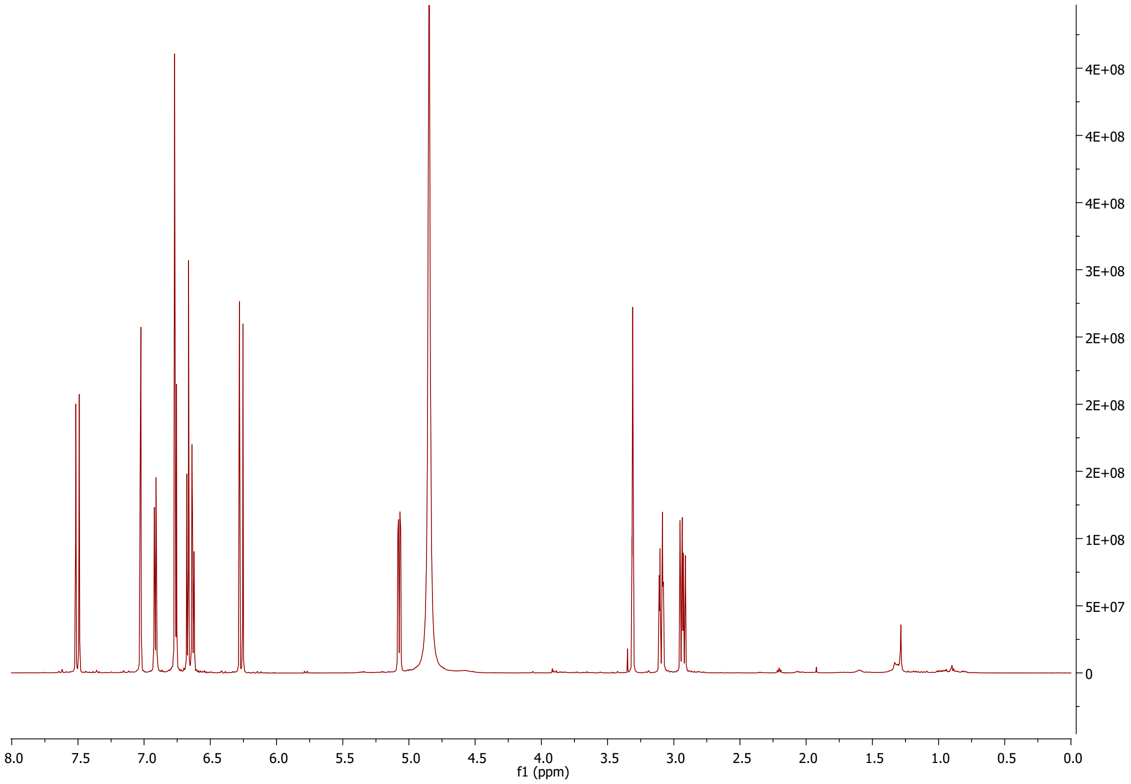

Figure S2: HSQC-NMR spectrum (600.19 MHz; 150.93 MHz) of rosmarinic acid in CD_3OD.

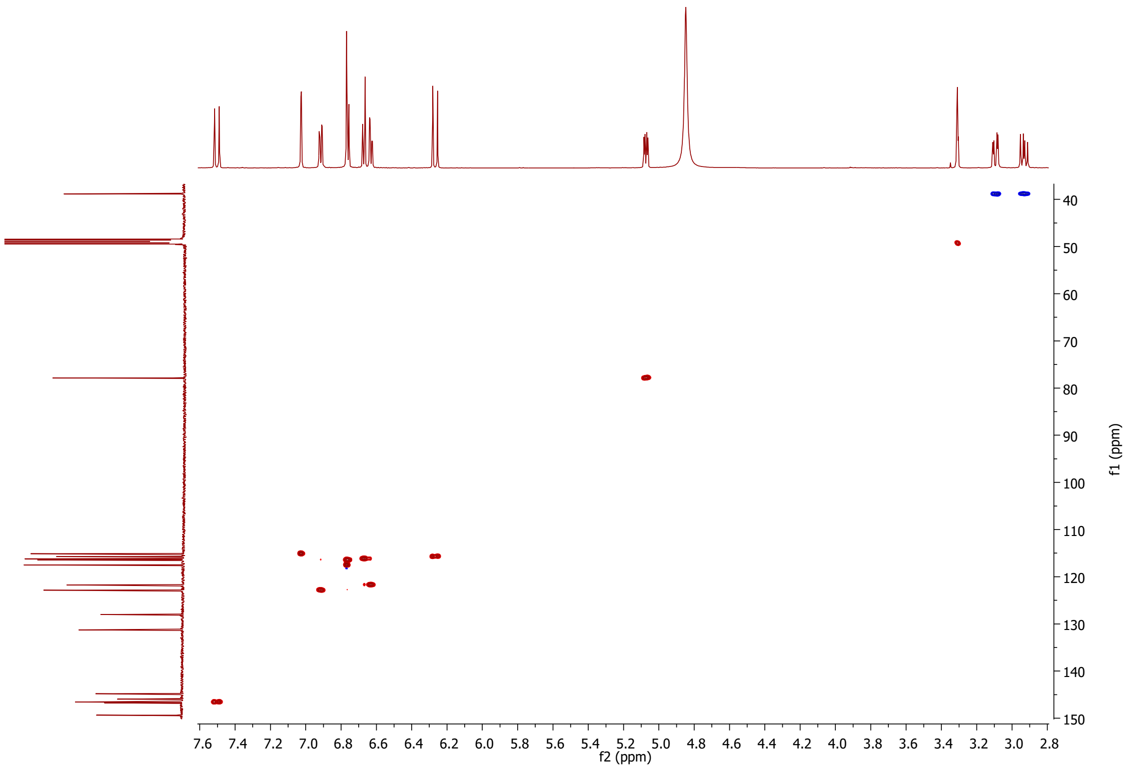

Figure S3: HMBC-NMR spectrum (600.19 MHz; 150.93 MHz) of rosmarinic acid in CD_3OD.

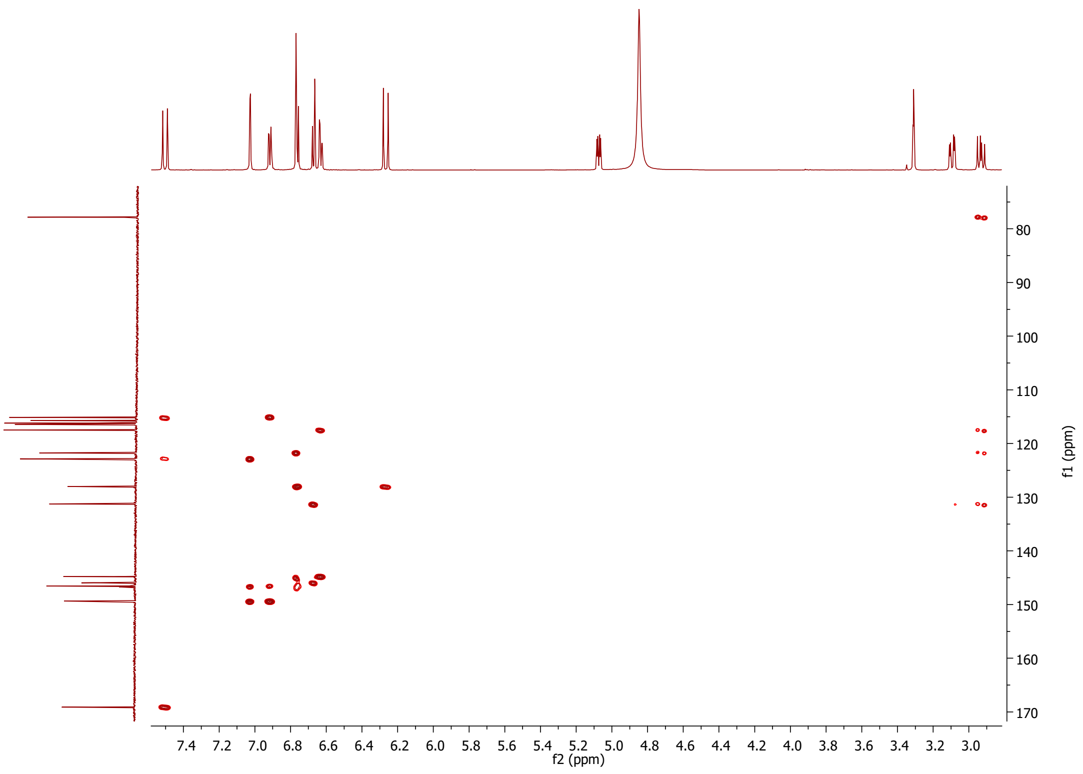

Figure S4: COSY-NMR spectrum (600.19 MHz) of rosmarinic acid in CD_3OD.

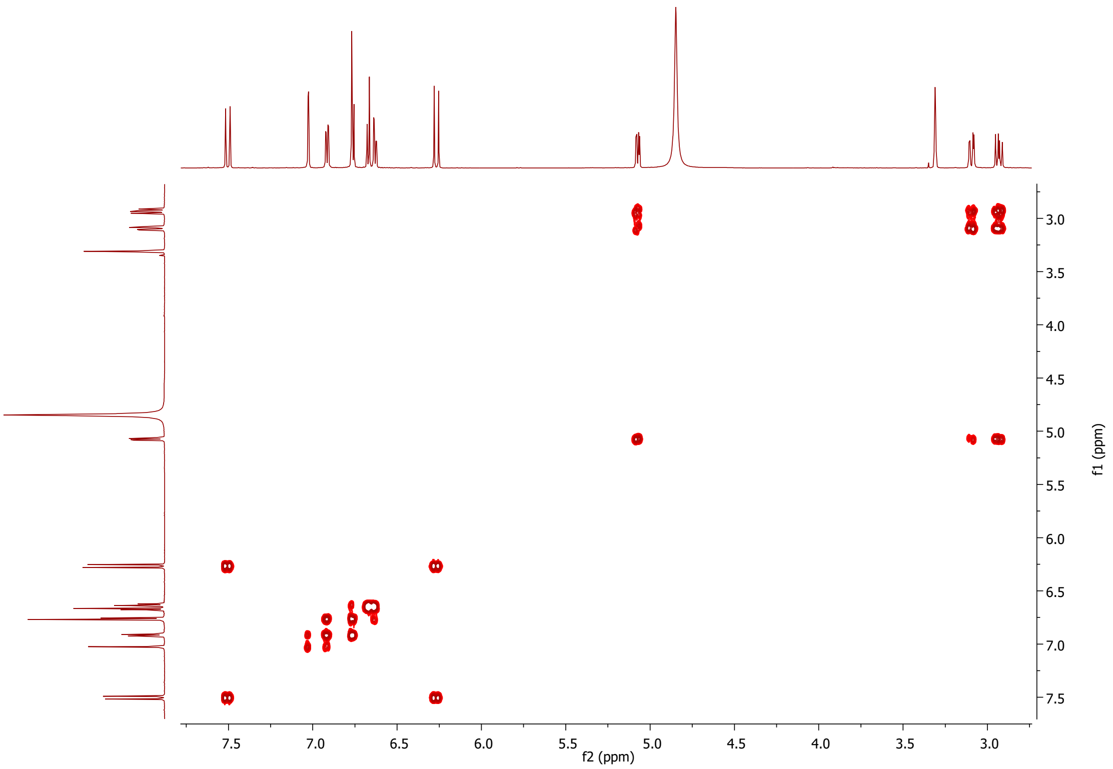

Supplement: Supplementary file 1 [file molecules-23-00653-s001.pdf]
